# Requirement of hepatic pyruvate carboxylase during fasting, high fat, and ketogenic diet

**DOI:** 10.1016/j.jbc.2022.102648

**Published:** 2022-10-28

**Authors:** Ebru S. Selen, Susana Rodriguez, Kyle S. Cavagnini, Han-Byeol Kim, Chan Hyun Na, Michael J. Wolfgang

**Affiliations:** 1 Department of Biological Chemistry, The Johns Hopkins University School of Medicine, Baltimore, MD, USA; 2 Department of Neurology, The Johns Hopkins University School of Medicine, Baltimore, MD, USA

**Keywords:** Pyruvate Carboxylase, fasting, gluconeogenesis, acetylation, mitochondria, BCA, bicinchoninic acid, BHB, β-hydroxybutyrate, cDNA, complementary DNA, FDR, false discovery rate, HFD, high-fat diet, IAP, immunoaffinity purification, LC, liquid chromatography, MS, mass spectrometry, NEFA, nonesterified fatty acid, OAA, oxaloacetate, TBST, Tris-buffered saline with Tween-20, TCA, tricarboxylic acid, TG, triglyceride

## Abstract

Pyruvate has two major fates upon entry into mitochondria, the oxidative decarboxylation to acetyl-CoA *via* the pyruvate decarboxylase complex or the biotin-dependent carboxylation to oxaloacetate *via* pyruvate carboxylase (Pcx). Here, we have generated mice with a liver-specific KO of pyruvate carboxylase (Pcx^L−/−^) to understand the role of Pcx in hepatic mitochondrial metabolism under disparate physiological states. Pcx^L−/−^ mice exhibited a deficit in hepatic gluconeogenesis and enhanced ketogenesis as expected but were able to maintain systemic euglycemia following a 24 h fast. Feeding a high-fat diet to Pcx^L−/−^ mice resulted in animals that were resistant to glucose intolerance without affecting body weight. However, we found that Pcx^L−/−^ mice fed a ketogenic diet for 1 week became severely hypoglycemic, demonstrating a requirement for hepatic Pcx for long-term glycemia under carbohydrate-limited diets. Additionally, we determined that loss of Pcx was associated with an induction in the abundance of lysine-acetylated proteins in Pcx^L−/−^ mice regardless of physiologic state. Furthermore, liver acetyl-proteomics revealed a biased induction in mitochondrial lysine-acetylated proteins. These data show that Pcx is important for maintaining the proper balance of pyruvate metabolism between oxidative and anaplerotic pathways.

The liver can exhibit dramatic metabolic shifts depending on nutritional and/or dietary state. This metabolic flexibility is most aptly demonstrated by the shift between *ad libitum* feeding and fasting where the liver becomes a net consumer or producer of blood glucose, respectively (1). Conversely, the liver is a net producer and then consumer of fatty acids between the carbohydrate replete and fasted states. This shift in macronutrient metabolism is accomplished by shifts in tricarboxylic acid (TCA) cycle flux whereby carbon is partitioned into either the reductive or oxidative branches of the TCA cycle to facilitate gluconeogenesis or fatty acid synthesis. While the fate of pyruvate is unique among the fed and fasted states, pyruvate carboxylase is central to both by generating oxaloacetate (OAA) from pyruvate (2).

Pyruvate entry into mitochondria is accompanied by the concomitant partitioning of pyruvate into two major fates, the oxidative decarboxylation to acetyl-CoA and CO_2_ *via* the pyruvate decarboxylase complex or its biotin-dependent carboxylation to OAA *via* pyruvate carboxylase (3). Pyruvate carboxylase contributes to both gluconeogenesis and fatty acid synthesis. Pyruvate carboxylase generates OAA as a mitochondrial-derived gluconeogenic substrate. Alternatively, pyruvate carboxylase generates OAA for the balanced synthesis of citrate by citrate synthase and is therefore highly expressed in lipogenic and steroidogenic tissues. Lipogenesis is a highly cataplerotic process and pyruvate carboxylase is a major mechanism by which the TCA cycle is replenished (4). Fatty acid oxidation is required for gluconeogenesis in part by generating ample mitochondrial acetyl-CoA to allosterically activate pyruvate carboxylase in addition to its roles in generating NADH, FADH2, and ATP. The activation of pyruvate carboxylase by acetyl-CoA derived from fatty acids has been proposed to play a major regulatory role in potentiating inappropriate gluconeogenesis in insulin resistance (5). Interfering with this activation could be a beneficial strategy in the treatment of type II diabetes (6, 7).

Here, we generated liver-specific pyruvate carboxylase KO mice in order to determine the requirements of hepatic pyruvate carboxylase in the liver and systemic metabolism following disparate nutritional and physiological states. We found that the liver-specific loss of pyruvate carboxylase was surprisingly well tolerated during a 24 h fast and even improved glucose tolerance during high fat feeding. However, a carbohydrate-limited ketogenic diet resulted in rapid metabolic decompensation. These data show the requirement of pyruvate carboxylase mediated anaplerosis in the liver under disparate metabolic conditions.

## Results

### Pyruvate carboxylase is required for hepatic gluconeogenesis but has a minimal impact on fasting blood glucose

**Figure 1:**
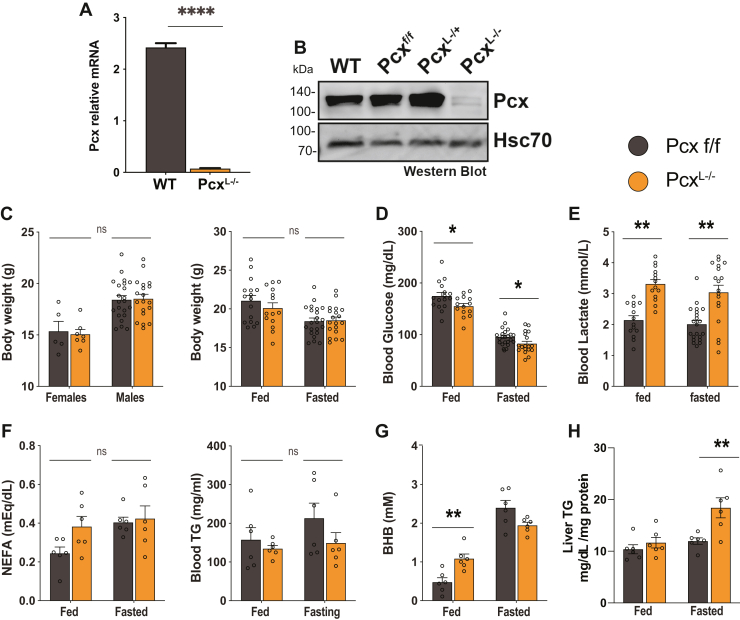
**Development of liver-specific pyruvate carboxylase KO mice.***A*, mRNA expression of Pcx in Pcx^f/f^ and Pcx^L−/−^ mice. *B*, Western blot of Pcx in Pcx^f/f^, Pcx^L−/+^, and Pcx^L−/−^ mice. *C*, body weight of 9-week-old male and female Pcx^f/f^ and Pcx^L−/−^ mice and 9-week-old fed and 24 h fasted male Pcx^f/f^ and Pcx^L−/−^ mice. *D*, blood glucose of 9-week-old fed and 24 h fasted male Pcx^f/f^ and Pcx^L−/−^ mice. *E*, blood lactate of 9-week-old fed and 24 h fasted male Pcx^f/f^ and Pcx^L−/−^ mice. *F*, blood NEFA and triglyceride of 9-week-old fed and 24 h fasted male Pcx^f/f^ and Pcx^L−/−^ mice. *G*, blood beta-hydroxybutyrate of 9-week-old fed and 24 h fasted male Pcx^f/f^ and Pcx^L−/−^ mice. *H*, liver triglyceride content of 9-week-old fed and 24 h fasted male Pcx^f/f^ and Pcx^L−/−^ mice. Data are expressed as mean ± SEM. ∗*p* < 0.05; ∗∗*p* < 0.01; ∗∗∗*p* < 0.001. NEFA, nonesterified fatty acid.

Upon fasting, pyruvate carboxylase is activated by acetyl-CoA derived from fatty acid ß-oxidation to generate OAA from pyruvate for hepatic gluconeogenesis. To better understand the requirements for Pcx in hepatic metabolism, we generated a hepatocyte-specific deletion of *pcx* in mice. Pcx transgenic mice from the European Mutant Mouse Archive (C57BL/6NTac-Pcx Tm1a) were obtained and bred to Flpe germline deleter mice (Jax #5705) to generate mice with Pcx that contain a floxed exon 10. These mice were then bred to Albumin-Cre mice to generate a hepatocyte-specific deletion of pyruvate carboxylase (Pcx^L−/−^) and littermate controls (Pcx^f/f^). The loss of Pcx in the liver of Pcx^L−/−^ mice was validated at the mRNA and protein level (Fig. 1, *A* and *B*). The loss of Pcx did not alter the body weight of male or female mice under fed or fasted conditions (Fig. 1*C*). The hepatic-specific loss of Pcx resulted in a statistically significant suppression of both fed and fasted blood glucose of Pcx^L−/−^ mice (Fig. 1*D*). However, this small suppression in blood glucose is not likely physiologically significant. The loss of Pcx did result in an increase in circulating lactate, consistent with human inborn errors of pyruvate carboxylase deficiency (OMIM # 266150) (8) (Fig. 1*E*). Pcx^L−/−^ mice exhibited normal circulating nonesterified fatty acid (NEFA) and triglycerides (TGs) (Fig. 1*F*). The ketone body β-hydroxybutyrate (BHB) was increased in fed Pcx^L−/−^ mice, suggesting a greater reliance on fatty acid oxidation as an energy source (Fig. 1*G*). Finally, liver TG content was increased in Pcx^L−/−^ mice following a 24 h fast consistent with an increased reliance on fatty acid catabolism (Fig. 1*H*). Taken together, the loss of hepatic pyruvate carboxylase resulted in mice that survive a 24 h fast with surprisingly physiologically normal glycemia.

**Figure 2:**
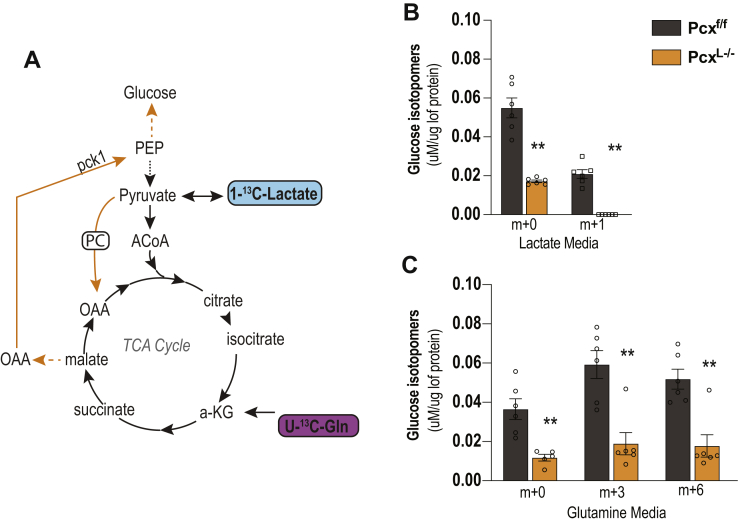
**Requirement of pyruvate carboxylase for hepatic gluconeogenesis**. *A*, experimental setup for tracing glutamine and lactate into glucose. *B*, glucose production in Pcx^f/f^ and Pcx^L−/−^ primary hepatocytes from 1-^13^C-lactate shows a suppression of gluconeogenesis. *C*, glucose production in Pcx^f/f^ and Pcx^L−/−^ primary hepatocytes from U-^13^C-glutamine shows a suppression of gluconeogenesis. Data are expressed as mean ± SEM by *t* test between genotypes ∗∗*p* < 0.01.

Given that Pcx^L−/−^ mice were able to maintain circulating fasting glucose concentrations, we next examined the requirement for pyruvate carboxylase in hepatic gluconeogenesis directly. We isolated primary hepatocytes from control and Pcx^L−/−^ mice. We then incubated the hepatocytes with 1-^13^C–labeled lactate or U-^13^C–labeled glutamine and measured the production of glucose from these substrates (Fig. 2*A*). The loss of pyruvate carboxylase suppressed the ability of hepatocytes to perform gluconeogenesis as the total production of glucose *in vitro* was suppressed (Fig. 2, *B* and *C*). Specifically, glucose production from lactate (Fig. 2*B*) or glutamine (Fig. 2*C*) was significantly suppressed, consistent with another pyruvate carboxylase loss of function model (9). In total, these results show that while pyruvate carboxylase is required for gluconeogenesis, other gluconeogenic tissues (*e*.*g*., kidney) can compensate for the loss of hepatic gluconeogenesis during a 24 h fast (10). These data show that despite a block in gluconeogenesis from mitochondria, mice demonstrate a remarkable systemic resiliency to fasting induced hypoglycemia.

**Figure 3:**
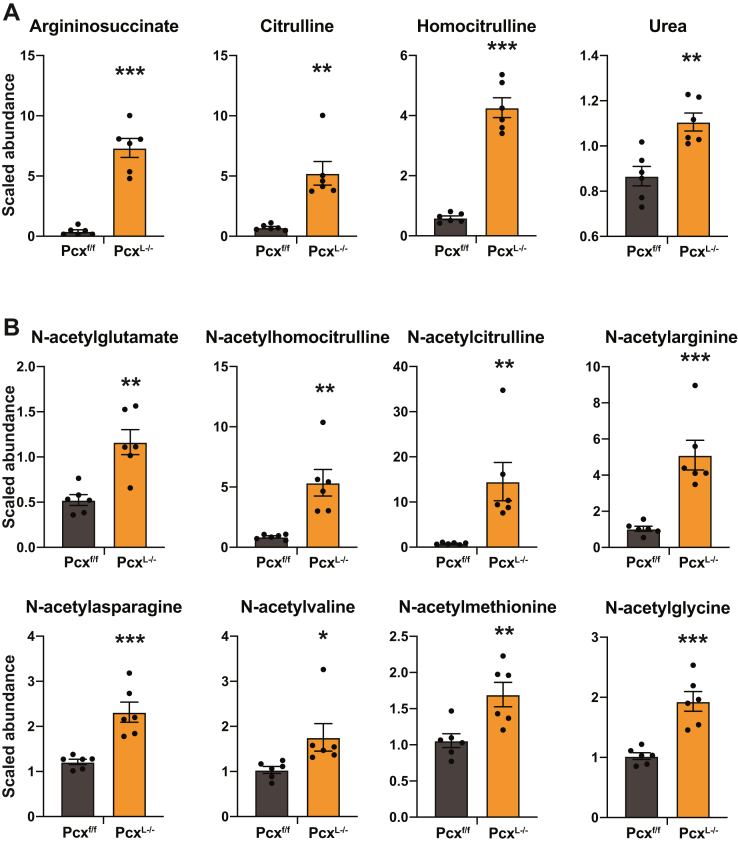
**Unbiased metabolomics from fasting liver-specific pyruvate carboxylase KO liver.***A*, urea cycle intermediates of 9-week-old fed and 24 h fasted male Pcx^f/f^ and Pcx^L−/−^ mice. *B*, N-acetylated amino acids from 9-week-old 24 h fasted male Pcx^f/f^ and Pcx^L−/−^ mice. Data are expressed as mean ± SEM. ∗*p* < 0.05; ∗∗*p* < 0.01; ∗∗∗*p* < 0.001.

We next turned to unbiased steady-state metabolomics to understand the effect of deleting hepatic pyruvate carboxylase on the liver metabolome following a 24 h fast (Table S2). The most over-represented class of metabolites in Pcx^L−/−^ liver were urea cycle intermediates. Argininosuccinate, citrulline, and homocitrulline were all significantly induced in Pcx^L−/−^ liver as well as urea (Fig. 3*A*). Consistent with the increase in urea cycle intermediates, N-acetylglutamate, a positive regulator of carbamoyl phosphate synthetase I was elevated (Fig. 3*B*) (11). Unexpectedly, almost all N-acetylated forms of free amino acids were elevated in Pcx^L−/−^ liver (Fig. 3*B*).

### The loss of hepatic pyruvate carboxylase protects mice from high-fat diet–induced glucose intolerance

**Figure 4:**
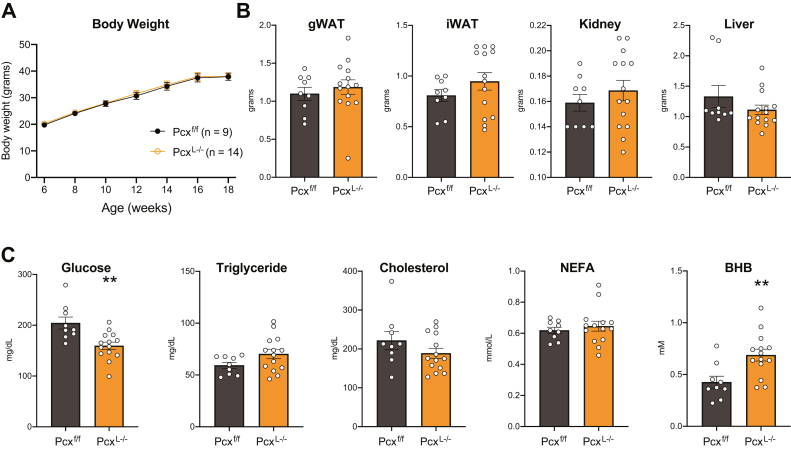
**The role of high fat feeding on liver-specific pyruvate carboxylase KO mice.***A*, body weight gain of male Pcx^f/f^ and Pcx^L−/−^ mice fed a high fat diet for 12 weeks *B*, weight of gonadal white adipose tissue (gWAT), inguinal (iWAT), kidney, and liver of male Pcx^f/f^ and Pcx^L−/−^ mice fed a high fat diet for 12 weeks *C*, blood glucose, triglyceride, cholesterol, NEFA, and beta-hydroxybutyrate in the fed state of male Pcx^f/f^ and Pcx^L−/−^ mice fed a high fat diet for 12 weeks. Data are expressed as mean ± SEM. ∗*p* < 0.05; ∗∗*p* < 0.01; ∗∗∗*p* < 0.001. NEFA, nonesterified fatty acid.

**Figure 5:**
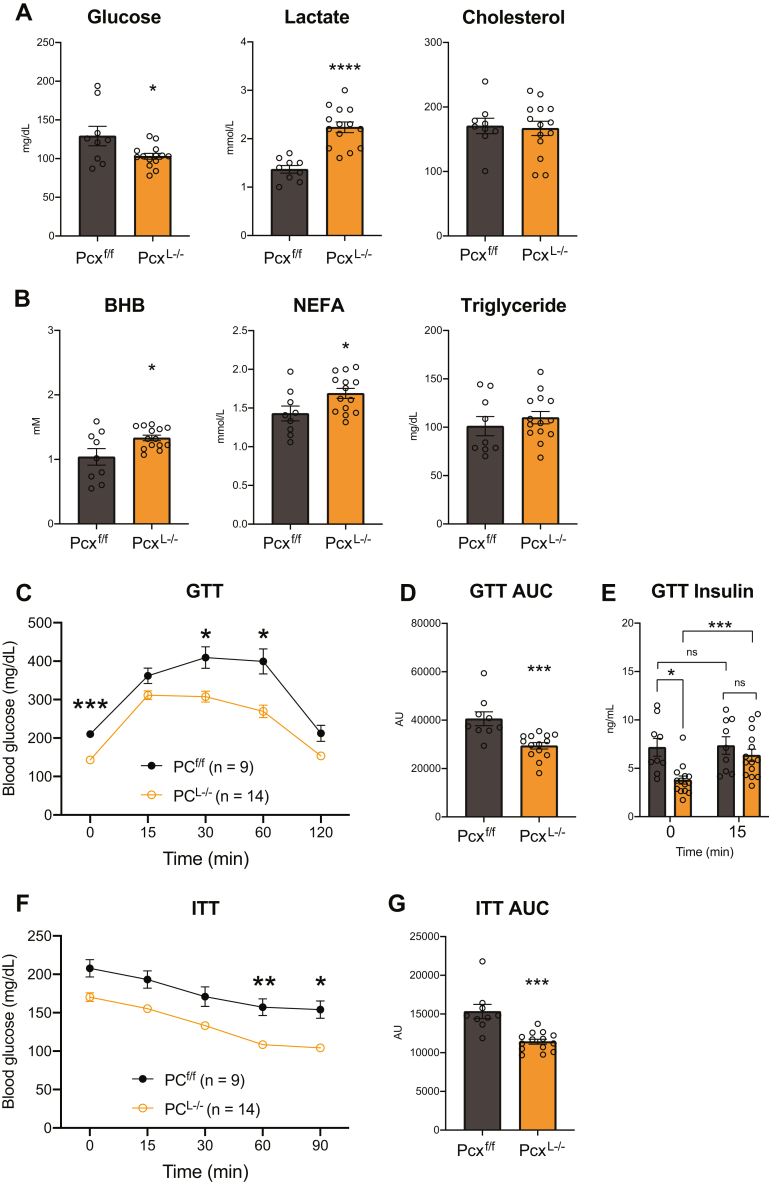
**Liver-specific pyruvate carboxylase KO mice are protected from high-fat diet–induced glucose intolerance**. *A*, fasted blood glucose, lactate, and cholesterol of male Pcx^f/f^ and Pcx^L−/−^ mice fed a high fat diet for 12 weeks. *B*, fasted blood beta-hydroxybutyrate (BHB), NEFA, and triglyceride of male Pcx^f/f^ and Pcx^L−/−^ mice fed a high fat diet for 12 weeks *C*, intraperitoneal glucose tolerance tests of male Pcx^f/f^ and Pcx^L−/−^ mice fed a high fat diet for 12 weeks. *D*, area under the curve (AUC) of glucose tolerance test of male Pcx^f/f^ and Pcx^L−/−^ mice fed a high fat diet for 12 weeks. *E*, fasted insulin before and 15 min after intraperitoneal glucose administration of male Pcx^f/f^ and Pcx^L−/−^ mice fed a high fat diet for 12 weeks *F*, insulin tolerance tests of male Pcx^f/f^ and Pcx^L−/−^ mice fed a high fat diet for 12 weeks. *G*, AUC of insulin tolerance test of male Pcx^f/f^ and Pcx^L−/−^ mice fed a high fat diet for 12 weeks. Data are expressed as mean ± SEM. ∗*p* < 0.05; ∗∗*p* < 0.01; ∗∗∗*p* < 0.001. NEFA, nonesterified fatty acid.

Inappropriate hepatic gluconeogenesis is thought to exacerbate hyperglycemia in insulin resistance and type II diabetes. To understand the role of pyruvate carboxylase and hepatic gluconeogenesis in insulin resistance, we placed male and female control and Pcx^L−/−^ mice on a high-fat diet (HFD) for 12 weeks. We did not observe any difference in body weight gain between control and Pcx^L−/−^ mice (Figs. 4*A* and S1*A*). Consistently, we did not observe differences in gonadal white adipose tissue, inguinal white adipose tissue, or liver weights (Figs. 4*B* and S1*B*). Female but not male Pcx^L−/−^ mice exhibited a significant increase in kidney weight (Fig. S1*B*). Male Pcx^L−/−^ mice exhibited a significant suppression in blood glucose while TGs, cholesterol, and NEFAs were not affected (Fig. 4*C*). Similar to chow fed Pcx^L−/−^ mice, BHB concentrations were significantly increased in HFD-fed male Pcx^L−/−^ mice (Fig. 4*C*). Female mice fed a HFD exhibited no changes in circulating metabolites (Fig. S1*C*). Fasting HFD-fed male and female Pcx^L−/−^ mice resulted in a suppression in blood glucose and increase in blood lactate concentrations (Figs. 5*A* and S2*A*). Fasting BHB and NEFA were increased in HFD-fed male and female Pcx^L−/−^ mice without affecting TG or cholesterol concentrations (Figs. 5*B* and S2*B*). Glucose tolerance tests revealed an improvement in blood glucose tolerance with increased glucose clearance and lower insulin levels in male HFD-fed Pcx^L−/−^ mice (Fig. 5, *C* and *D*). Glucose-invoked insulin production was also improved (Fig. 5*E*). Insulin tolerance tests revealed an improvement in insulin-stimulated blood glucose clearance in male HFD-fed Pcx^L−/−^ mice (Fig. 5, *F* and *G*). Female HFD-fed Pcx^L−/−^ mice did not exhibit changes in glucose or insulin tolerance (Fig. S2, *C*–*F*). These data show that inhibiting hepatic pyruvate carboxylase accelerates hepatic ketogenesis and improves diet-induced glucose intolerance in a somewhat sexually dimorphic manner.

### Pcx^L−/−^ mice are unable to sustain glucose homeostasis under a ketogenic diet

**Figure 6:**
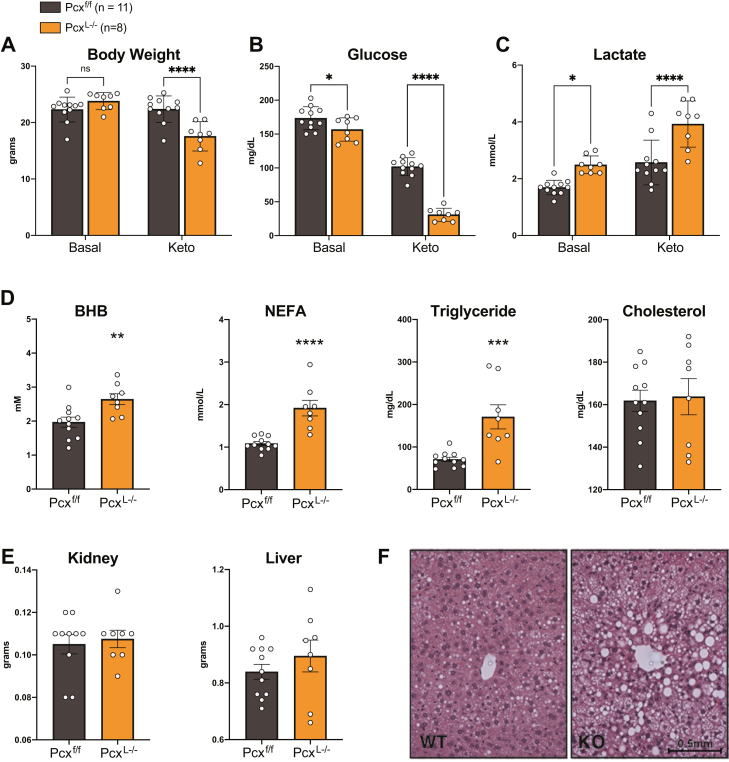
**A ketogenic diet causes metabolic decompensation in liver-specific pyruvate carboxylase KO mice**. *A*, body weight of 9-week-old male Pcx^f/f^ and Pcx^L−/−^ mice before and after feeding a ketogenic diet for 1 week. *B*, blood glucose of 9-week-old male Pcx^f/f^ and Pcx^L−/−^ mice before and after feeding a ketogenic diet for 1 week. *C*, blood lactate of 9-week-old male Pcx^f/f^ and Pcx^L−/−^ mice before and after feeding a ketogenic diet for 1 week. *D*, blood beta-hydroxybutyrate (BHB), NEFA, triglyceride, and cholesterol of 9-week-old male Pcx^f/f^ and Pcx^L−/−^ mice after feeding a ketogenic diet for 1 week *E*, kidney and liver weights of 9-week-old male Pcx^f/f^ and Pcx^L−/−^ mice after feeding a ketogenic diet for 1 week. *F*, H&E staining of liver from male Pcx^f/f^ and Pcx^L−/−^ mice after feeding a ketogenic diet for 1 week. Data are expressed as mean ± SEM. ∗*p* < 0.05; ∗∗*p* < 0.01; ∗∗∗*p* < 0.001. NEFA, nonesterified fatty acid.

Pcx^L−/−^ mice tolerated a 24 h fast and HFD feeding with normal and improved glucose homeostasis, respectively. Therefore, we next tested the ability of male and female Pcx^L−/−^ mice to maintain glucose homeostasis under a carbohydrate restricted ketogenic diet. Before administering the diet, Pcx^L−/−^ and control mice had equal body weights and a mild suppression of blood glucose and increased blood lactate consistent with previous chow-fed cohorts. However, after 7 days of ketogenic diet feeding, male and female Pcx^L−/−^ mice lost considerable body weight and exhibited metabolic decompensation with exacerbated differences in blood glucose and lactate concentrations (Figs. 6, *A*–*C* and S3, *A*–*C*). While chow-fed mice exhibited a statistically significant yet physiologically mild suppression in blood glucose, 7 days of a ketogenic diet resulted in a physiologically significant moribund circulating blood glucose. Furthermore, ketogenic diet increased BHB, NEFA, and TGs in male Pcx^L−/−^ mice compared to littermate controls (Figs. 6*D* and S3*D*). We also measured circulating levels of BHB, NEFA, and TGs in Pcx^L−/−^ female mice following exposure to ketogenic diet. With the exception of significantly increased NEFA levels, Pcx^L−/−^ female mice displayed no further alterations to serum metabolites (Fig. S3*D*). Tissue weights of kidney and liver were not different in male and female mice, but the livers of Pcx^L−/−^ male mice were visibly lipid laden with abundant lipid droplet deposition (Figs. 6, *E*, *F* and S3*E*). These data demonstrate the requirement of hepatic pyruvate carboxylase for long term glycemic control during carbohydrate-limited conditions.

### RNA-seq analysis of pyruvate carboxylase KO liver

**Figure 7:**
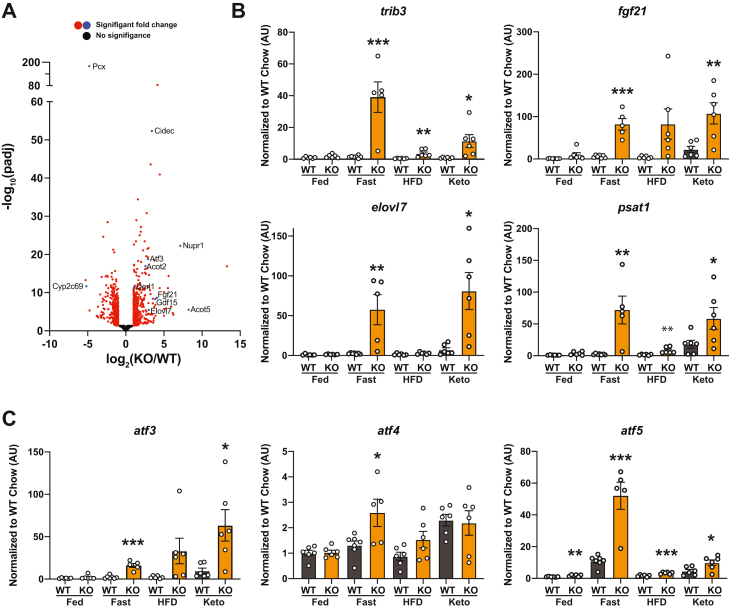
**Effect of diet on transcript abundance in liver-specific pyruvate carboxylase KO mice.***A*, volcano plot of differential transcript abundance from RNA-seq comparing 24 h fasted Pcx^f/f^ and Pcx^L−/−^ liver (n = 4). *B*, qPCR analysis of *trib3*, *fgf21*, *elovl7*, and *psat1* comparing fed, 24 h fasted, 12 HFD fed, or 1 week ketogenic diet fed Pcx^f/f^ and Pcx^L−/−^ liver (n = 5–7). Data are expressed as mean ± SEM. ∗*p* < 0.05; ∗∗*p* < 0.01; ∗∗∗*p* < 0.001. *C*, qPCR analysis of *aft3*, *atf4*, and *atf5* comparing fed, 24 h fasted, 12 HFD fed, or 1 week ketogenic diet fed Pcx^f/f^ and Pcx^L−/−^ liver (n = 5–7). Data are expressed as mean ± SEM. ∗*p* < 0.05; ∗∗*p* < 0.01; ∗∗∗*p* < 0.001. HFD, high-fat diet; qPCR, quantitative PCR.

In order to examine the broad gene expression response of Pcx^L−/−^ mice to fasting, we performed RNA-seq on liver from 24 h fasted control and Pcx^L−/−^ mice (Tables 1 and S3). Out of ∼22,000 transcripts annotated, over 3900 transcripts were significantly altered in Pcx^L−/−^ mice with 437 induced and 397 suppressed by at least 2-fold (Fig. 7*A*). Gene ontology analysis of significantly altered transcripts did not identify meaningful overarching patterns within the data. However, there were transcripts of interest altered within the dataset. For example, *trib3* was greater than 17-fold higher in Pcx^L−/−^ mice and significantly elevated in Pcx^L−/−^ liver in the fasted, HFD, and ketogenic diet–fed states. While *trib3* has been implicated in hepatic insulin signaling and fatty acid synthesis, its precise role and requirement in glucose and lipid homeostasis has been less clear (12, 13). A subset of putative Pparα responsive transcripts was upregulated including *fgf21*, *acot2*, *acot5*, and *elovl7*. The *fgf21*, *elovl7*, and *psat1* transcripts were also induced in the fasted and ketogenic diet–fed states (Fig. 7*B*). Finally, the stress responsive *atf3, atf4*, and *atf5* were higher in fasted Pcx^L−/−^ liver (Fig. 7*C*). While *atf4* was moderately elevated in Pcx^L−/−^ liver only in the fasted state, *atf3* and *atf5* were elevated in the fasted, HFD, and ketogenic diet–fed states (Fig. 7*C*). Relevant to the present study, Atf5 has been implicated in the mammalian mitochondrial stress response (14). These data suggest that Pcx^L−/−^ liver is experiencing metabolically induced cell stress particularly when carbohydrates are restricted.

**Table 1:** Selected transcripts from RNA-seq differential expression analysis of 24 h fasted male control and Pcx^L−/−^ liver that were changed by more than 2-fold (n = 4/group)

Gene	Protein	Pcx^L-/-^	Pcx^f/f^	log2 fold	p-adj
Psat1	Phosphoserine aminotransferase 1	404	18	3.91	1.91E-44
Trib3	Tribbles pseudokinase 3	4846	271	3.91	2.25E-84
Cidec	Cell death–inducing DFFA-like effector c	2988	275	3.22	4.02E-53
Cyb5r1	Cytochrome b5 reductase 1	1239	128	3.04	1.54E-44
Atf3	Activating transcription factor 3	96	12	2.62	4.84E-21
Pmm1	Phosphomannomutase 1	642	74	2.6	2.21E-15
Fgf21	Fibroblast growth factor 21	590	31	2.55	7.32E-09
Gdf15	Growth differentiation factor 15	628	38	2.5	1.29E-08
Acot2	Acyl-CoA thioesterase 2	2201	357	2.34	1.17E-17
Elovl7	ELOVL family member 7, elongation of long chain fatty acids	61	6	2.08	2.25E-06
Asns	Asparagine synthetase	450	87	1.99	5.96E-09
Apoa4	Apolipoprotein A-IV	49,370	12,511	1.89	1.22E-26
Acot6	Acyl-CoA thioesterase 6	119	28	1.87	6.17E-11
Atf5	Activating transcription factor 5	17,936	4976	1.78	9.51E-26
Acsl4	Acyl-CoA synthetase long-chain family member 4	3671	1070	1.64	1.03E-11
Sgk1	Serum/glucocorticoid regulated kinase 1	2818	642	1.62	5.43E-05
Slc7a1	Solute carrier family 7 (cationic amino acid transporter, y+ system), member 1	107	34	1.48	1.83E-07
Plin2	Perilipin 2	13,621	4934	1.36	6.75E-09
Fabp5	Fatty acid binding protein 5, epidermal	145	52	1.34	3.87E-08
Vldlr	Very low density lipoprotein receptor	340	128	1.33	6.67E-09
Cyp2c68	Cytochrome P450, family 2, subfamily c, polypeptide 68	1606	4202	−1.26	1.97E-06
Cyp2c39	Cytochrome P450, family 2, subfamily c, polypeptide 39	61	205	−1.37	0.000415
Cyp7b1	Cytochrome P450, family 7, subfamily b, polypeptide 1	510	1544	−1.39	7.66E-06
Ces1b	Carboxylesterase 1B	830	2460	−1.45	1.36E-09
Cyp2c70	Cytochrome P450, family 2, subfamily c, polypeptide 70	199	791	−1.45	0.000617
Etnppl	Ethanolamine phosphate phospholyase	1649	6514	−1.49	0.000291
Acss3	Acyl-CoA synthetase short-chain family member 3	175	576	−1.59	2.50E-11
Cyp2c67	Cytochrome P450, family 2, subfamily c, polypeptide 67	1004	3210	−1.61	6.13E-22
Ces3b	Carboxylesterase 3B	1651	5728	−1.64	1.47E-10
Cyp2c29	Cytochrome P450, family 2, subfamily c, polypeptide 29	1875	7001	−1.65	1.38E-07
Cyp2c37	Cytochrome P450, family 2. subfamily c, polypeptide 37	1394	5288	−1.74	5.19E-11
Cyp2c50	Cytochrome P450, family 2, subfamily c, polypeptide 50	1908	10,077	−2.09	7.38E-12
Cyp2c38	Cytochrome P450, family 2, subfamily c, polypeptide 38	310	1694	−2.18	7.07E-15
Cyp2c54	Cytochrome P450, family 2, subfamily c, polypeptide 54	923	5334	−2.24	4.24E-15
Pcx	Pyruvate carboxylase	674	18,859	−4.62	6.00E-181

### The loss of pyruvate carboxylase results in the increased abundance of lysine-acetylated mitochondrial proteins in the liver

**Figure 8:**
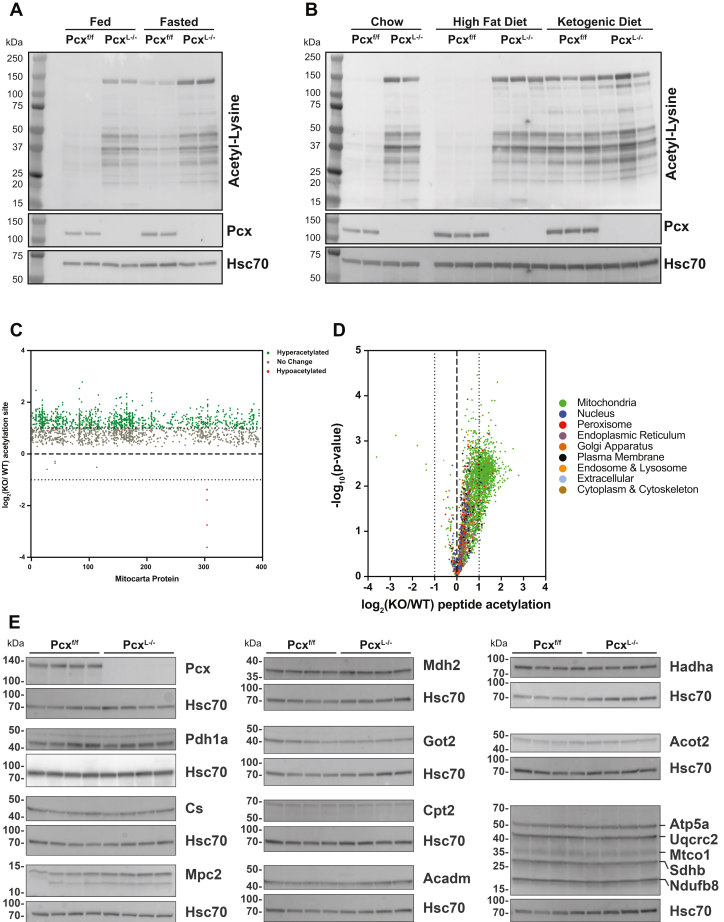
**The loss of pyruvate carboxylase elicits mitochondrial protein hyperacetylation.***A*, Western blot of acetylated proteins from 9-week-old fed and 24 h fasted Pcx^f/f^ and Pcx^L−/−^ mice. *B*, Western blot of acetylated proteins from 9-week-old fed, 24 h fasted, 12 week high fat diet–fed, and 1 week ketogenic diet–fed Pcx^f/f^ and Pcx^L−/−^ mice. *C*, quantitative TMT acetyl-proteomics of 24 h fasted Pcx^f/f^ and Pcx^L−/−^ livers showing mitochondrial proteins (n = 5). *D*, quantitative TMT acetyl-proteomics of 24 h fasted Pcx^f/f^ and Pcx^L−/−^ livers showing all proteins (n = 5). *E*, Western blots of mitochondrial proteins from 9-week-old Pcx^f/f^ and Pcx^L−/−^ mice (n = 4). Pyruvate Carboxylase (Pcx), Pyruvate Dehydrogenase 1a (Pdh1a), Citrate Synthase (Cs), Malate Dehydrogenase 2 (Mdh2), Mitochondrial Pyruvate Carrier 2 (Mpc2), Glutamine Oxaloacetate Transaminase 2 (Got2), Carnitine Palmitoyltransferase 2 (Cpt2), Acyl-CoA Dehydrogenase Medium Chain (Acadm), Hydroxyacyl-CoA Dehydrogenase Trifunctional Multienzyme Complex Subunit Alpha (Hadha), Acyl-CoA Thioesterase 2 (Acot2), ATP synthase, H+ transporting, mitochondrial F1 complex, alpha subunit 1 (Atp5a), ubiquinol cytochrome c reductase core protein 2 (Uqcrc2), mitochondrially encoded cytochrome c oxidase I (Mtco1), succinate dehydrogenase complex, subunit B (Sdhb), NADH:ubiquinone oxidoreductase subunit B8 (Ndufb8).

The metabolic changes we observed in the liver of Pcx^L−/−^ mice prompted us to investigate protein lysine acetylation more globally in Pcx^L−/−^ liver. Western blotting using antiprotein lysine antibodies revealed an increase in the abundance of lysine-acetylated proteins compared to WT littermates in both the fed and fasting states (Fig. 8*A*). Indeed, fed Pcx^L−/−^ liver exhibited higher abundance of lysine-acetylated proteins than even 24 h fasted control liver. Pcx^L−/−^ mice fed a HFD also exhibited a large increase in the abundance of lysine-acetylated proteins comparable to the increase observed by chow feeding (Fig. 8*B*). However, ketogenic diet–fed Pcx^L−/−^ and control mice exhibited a similar pattern of lysine-acetylated proteins, suggesting that the increase in lysine acetylation is associated with lipid oxidation. To gain a more granular view of the proteins with an increase in abundance of lysine-acetylated proteins, we mapped and quantified the differential abundance of lysine acetylated proteins *via* tandem mass tag (TMT)-based mass spectrometry (MS) (Table 2). We found that the abundance of lysine-acetylated proteins in mitochondria was greatly increased in the livers of Pcx^L−/−^ mice, consistent with altered mitochondrial metabolism in these mice (Fig. 8*C*). Interestingly, when analyzing the whole proteome, it is clear that mitochondrial protein hyperacetylation is the dominant signature in Pcx^L−/−^ liver with minimal changes in acetylpeptide levels outside of mitochondria (Fig. 8*D* and Table S4). To confirm the expression of these proteins was unchanged by the loss of Pcx, we performed Western blot analysis on 15 mitochondrial proteins in the livers of control and Pcx^L−/−^ liver. None of the protein concentrations we probed were changed even though they were the most highly differentially lysine-acetylated proteins. These data demonstrate that the loss of hepatic pyruvate carboxylase is associated with a dramatic increase in the abundance of lysine-acetylated proteins in mitochondria in the liver, suggesting additional metabolically driven posttranslational regulatory mechanisms.

**Table 2:** Selected acetylated peptides from TMT acetyl-proteomics of 24 h fasted male control and Pcx^L−/−^ liver (n = 5/group)

## Discussion

Pyruvate, derived largely from glycolysis, alanine transamination, and lactate conversion, proceeds *via* two major fates once it enters the mitochondrial matrix. To contribute to the oxidative TCA cycle, it is oxidatively decarboxylated by the pyruvate dehydrogenase complex generating acetyl-CoA and CO_2_. In this way, pyruvate can efficiently energize mitochondria and contribute to cellular ATP production. The other major fate for pyruvate is the biotin-dependent carboxylation to OAA *via* pyruvate carboxylase. Pyruvate carboxylase is uniquely important for hepatic TCA cycle function in both the fed and fasted states by providing an anaplerotic source for gluconeogenesis and fatty acid biosynthesis. Here, we have removed pyruvate carboxylase specifically in the liver to better understand the contribution of pyruvate mediated anaplerosis to liver and systemic metabolism. We have shown that the liver requires pyruvate carboxylase for gluconeogenesis, but its loss is well tolerated following a 24 h fast with a surprising metabolic resiliency. In fact, the suppression in hepatic gluconeogenesis protects Pcx^L−/−^ mice from HFD-induced glucose intolerance. However, longer term feeding of carbohydrate depleted diets to Pcx^L-/-^ mice results in their rapid metabolic decompensation.

The majority of pyruvate is transported into the mitochondrial matrix *via* the Mitochondrial Pyruvate Carrier (Mpc) located in the mitochondrial inner membrane (15, 16). The loss of either of the components of the Mpc, Mpc1, or Mpc2 is not sufficient to fully block mitochondrial pyruvate metabolism as pyruvate can be generated by multiple compensatory pathways such as alanine transamination or supported by glutamine anaplerosis (17, 18, 19). There is no such alternative pathway to compensate for the loss of pyruvate carboxylase. Surprisingly, we found that pyruvate carboxylase was uniquely required for gluconeogenesis and other substrates such as glutamine could not support gluconeogenesis in the absence of pyruvate carboxylase, consistent with the work of others (9). The biochemical rationale for why a KO of pyruvate carboxylase would be required for other sources of anaplerosis is not clear.

Fatty acid oxidation and pyruvate carboxylase activity are intimately connected. During fasting or starvation, fatty acids are oxidized in mitochondria to provide hepatocytes with ATP and NADH to facilitate gluconeogenesis and the carbon substrate (acetyl-CoA) for ketogenesis. This enables the liver to buffer blood glucose and provide alternative fuel (ketone bodies) for highly oxidative tissues during food deprivation. The loss of pyruvate carboxylase was associated with increased ketogenesis, suggesting their livers are likely relying on alternative fuels. People with disparate inborn errors in fatty acid oxidation exhibit life-threatening hypoketotic hypoglycemic bouts following a fast. Fatty acids cannot contribute to a net increase in the carbon skeleton of glucose during gluconeogenesis in mammals. One of the important canonical roles of hepatic fatty acid oxidation is to enable gluconeogenesis. It does this in large part by providing acetyl-CoA to allosterically activate pyruvate carboxylase. Previously, we characterized a conditional loss of function of liver *Cpt2* (Cpt2^L−/−^) mouse model with a lack of hepatic fatty acid oxidation and showed that they also exhibited a defect in hepatic gluconeogenesis but exhibited minimally altered glycemia following a 24 h fast (20). Also, similar to Pcx^L−/−^ mice, animals with a defect in hepatic fatty acid oxidation exhibited a rapid metabolic decompensation following a ketogenic diet at a similar timescale (20). The similarities between the phenotypes of mice with liver-specific deficits in pyruvate carboxylase and fatty acid oxidation support their intimate connection.

The liver-specific loss of glucose-6-phosphatase results in a similar phenotype as PcxL−/− mice including fasting euglycemia with increased ketogenesis (21, 22). This suggests that hepatic glucose production from non-mitochondrial sources such as glycerol or glycogen are also not required to maintain fasting glycemia and that mammals possess a remarkable resiliency to fasting hypoglycemia. This resiliency is likely mediated by the sensing of metabolic stress. RNA-seq analysis of Pcx^L−/−^ mice demonstrated a transcriptional response indicative of a mitochondrial stress response including the induction of transcripts such as *atf3*, *4*, and *5*. These changes were minimally effected in the fed state but upon metabolic challenge like fasting or high fat feeding, they increased sometimes dramatically. These results demonstrate a sophisticated mechanism for sensing and responding to metabolic stress.

Pcx^L−/−^ mice as well as Cpt2^L−/−^ mice with a defect in hepatic fatty acid oxidation are resistant to diet-induced glucose intolerance (23). Insulin resistance results in two seemingly opposing phenomenon in the liver. Insulin-resistant individuals exhibit an inability to repress gluconeogenesis during carbohydrate replete conditions. Also, insulin-resistant livers promote *de novo* lipogenesis, although insulin is a powerful activator of fatty acid synthesis. These observations are seemingly at odds and have been termed selective insulin resistance (24). The long-sought mechanism of this selective insulin signaling has been elusive. An alternative hypothesis suggests that the liver remains insulin sensitive and therefore promotes insulin-dependent *de novo* lipogenesis and that increased gluconeogenesis is mediated by activation of pyruvate carboxylase by acetyl-CoA derived from fatty acid oxidation (5). Therefore, insulin resistance in the adipose and the inability to suppress lipolysis and fatty acid delivery to the liver may play a predominant noncell autonomous role in promoting gluconeogenesis.

Livers from Pcx^L−/−^ mice exhibit a dramatic increase in the abundance of lysine-acetylated proteins. The increase in N-acetylated amino acids, ketogenesis, and mitochondrial lysine-acetylated proteins all suggest an increase in matrix acetyl-CoA. Increased hepatocyte fatty acid oxidation is likely responsible as the lysine-acetylated proteins can be normalized by feeding Pcx^L−/−^ mice a ketogenic diet. Although much has been written regarding the posttranslational regulation exerted by mitochondrial lysine acetylation, a more detailed analysis suggests that mitochondrial lysine acetylation/deacetylation plays a limited role in regulating mitochondrial energetics (25, 26, 27, 28, 29). There has been no definitive mitochondrial lysine acetyltransferase identified and the phenotypes of the major deacetylates have been inconsistent (29). The role of mitochondrial lysine acetylation is therefore not clear and may not play a protein and site-specific regulatory role as previously envisioned but may be an excellent indicator of long term mitochondrial metabolic state analogous to glycated hemoglobin.

Here, we have demonstrated the requirements of pyruvate carboxylase under disparate nutritional states. Pyruvate carboxylase represents a major pathway for mitochondrial pyruvate metabolism. While the fate of pyruvate is unique among the fed and fasted states, pyruvate carboxylase contributes to both. While the loss of pyruvate carboxylase in the liver is tolerated during a 24 h fast and is protective for high fat–induced glucose intolerance, a ketogenic diet causes rapid metabolic decompensation. These data show the central requirement for hepatic pyruvate anaplerosis.

## Experimental procedures

Pyruvate carboxylase (Pcx) transgenic mice were obtained from the European Mutant Mouse Archive (C57BL/6NTac-Pcx Tm1a) and bred to Flpe germline deleter mice (Jax #5705) to obtain mice with Pcx that contain a floxed exon 10. Genotyping primers for the floxed allele were forward, 5′-CACACTGGCCTAAAGCTTG-3′, and reverse, 5′- TTCCTGACTCCCTATGACAC-3’. The size of the amplified PCR product was 387 bp. These mice were then bred to Albumin-Cre mice to generate a hepatocyte-specific deletion of pyruvate carboxylase (Pcx^L−/−^) and littermate controls (Pcx^f/f^). Genotyping primers for Cre were forward, 5′-CCAGCTAAACATGCTTCATCG-3′, and reverse, 5′-CTAACCAGCGTTTTCGTTCTG-3’. The size of the amplified product was 336 bp. All mice were housed in a facility with ventilated racks on a 14 h light/10 h dark cycle with *ad libitum* access to a standard rodent chow (18% protein, 2018SX, Envigo Teklad Diets). Additional cohorts of male and female Pcx^L−/−^ mice and their littermate controls received access to either a HFD (60% kcal derived from fat, #D12492, Research Diets) at 6 weeks of age for 3 months or a ketogenic diet (Keto; 8.6% protein, 75.1% fat, 3.2% carbohydrate, AIN-76A-Modified, High Fat, Paste; F3666, BioServ) at 9 weeks of age for 1 week. Food deprivation studies were performed on 9-week-old mice for 24 h (3 PM.-3 PM) or 4 h (11 AM-3 PM) for fed state. Tissues and serum were collected and flash-frozen at time of harvest. All procedures were performed in accordance with the NIH’s Guide for the Care and Use of Laboratory Animals and under the approval of the Johns Hopkins School of Medicine Animal Care and Use Committee.

### Glucose and insulin tolerance test

Pcx^L−/−^ and Pcx^f/f^ mice were fasted 6 h before being injected intraperitoneally with a glucose solution in saline at a dose of 1 mg/g body weight. Blood glucose was measured at 0, 15, 30, 60, and 120 min post glucose injection using a glucometer (Nova Biomedical). Blood was collected from the tail vein using capillary collection tubes (Microvette CB 300, Sarstedt) before and 15 min post injection to assess insulin levels. Serum was isolated from blood and stored at −80 °C. Serum insulin levels were measured using an ELISA kit (Millipore). For insulin tolerance tests, mice were fasted for 2 h before being injected intraperitoneally with human recombinant insulin (catalog no.: # 12585014, Gibco for Thermo Fisher Scientific) at a dose of 1 unit/kg body weight. Blood glucose was measured at 0 and 15, 30, 60, and 90 min post insulin injection using a glucometer (Nova Biomedical).

### Histology

Livers from Pcx^L−/−^ and Pcx^f/f^ mice (n = 3/biological replicates per genotype) were immediately fixed in 10% neutral buffered formalin following dissection and stored at 4 °C. Fixed liver tissues were embedded in paraffin, sectioned, and stained for H&E at the Histology Reference Laboratory at The Johns Hopkins University School of Medicine.

#### Primary hepatocyte gluconeogenesis

Primary mouse hepatocytes were isolated from 8- to 10-week-old mice based on a previously published protocol (30, 31). After hepatocytes were isolated, they were resuspended in 15 ml complete culture medium, Media 199 with 10% fetal bovine serum (Gibco and Atlanta Biologicals), and 1% Pen/Strep and counted by trypan blue method using a hemocytometer to determine live cell counts and cell viability. About 19,000,000 live hepatocytes were isolated. Next, 3 to 5 × 10^5^ live hepatocytes were plated per well in 6-well plates precoated with collagen I (Gibco by Life Technologies) in 2 ml culture media. Hepatocytes were incubated for 24 h before treatment at 37 °C at 5% CO_2_. For ^13^C experiments, hepatocytes were washed with 1× PBS pH 7.4 (Quality Biological) to remove the overnight media. Hepatocytes were incubated in 500 nM dexamethasone, 100uM cAMP (#A3262, Sigma–Aldrich), 0.5% bovine serum albumin in glucose, glutamine, and phenol red–free Dulbecco's modified Eagle's medium for 1 h, supplemented with either 2 mM U-^13^C-glutamine (# 605166, Sigma–Aldrich) or 1-^13^C-Lactate (#CLM-1577-PK, Cambridge Isotope Laboratory, Inc). At the end of the incubation period, cells were prepped for metabolic extraction by quenching with 1 ml ice cold methanol, scraped, and transferred into microcentrifuge tubes. Next, 0.5 ml of ultrapure molecular grade water (Quality Biological) was added to each tube, vortexed for 30 s, and incubated at −20 °C, for 30 min to induce protein precipitation. Following the incubation, cells were centrifuged at 4 °C, at 13,000*g* for 10 min. The supernatant was collected to a new tube and dried overnight by speedvac. The pellets that contained the precipitated proteins were then incubated in 0.5 M NaOH overnight to solubilize the proteins. The following day, the protein content of each sample was calculated using the Pierce bicinchoninic acid (BCA) assay (Thermo Fisher Scientific) and used for determining protein concentrations for normalization purposes.

Dried samples were reconstituted with 50 μl 50:50 acetonitrile:water, vortexed, and centrifuged at 13,000*g* for 5 min. Next, 10 μl of the supernatant were transferred to a liquid chromatography (LC) vial for LC-triple quadrupole mass spectrometer (LCMSMS) analysis of glucose. LC separation was performed on Shimadzu Nexera XR UPLC system (Shimadzu). A UPLC BEH Amide, 2.1 × 100 mm, 1.7 μm column is used for the analysis (Waters). Optimal running conditions are as follows: 20 min equilibration time, 0 to 0.2 min 0%, 0.2 to 5 min 60%, 5 to 6 min 60%, 6 to 10 min 0%, flow rate: 0.2 ml/min, mobile phase A: 0.1% ammonium hydroxide in 80% acetonitrile: 20% water, mobile phase B: 0.1% ammonium hydroxide in 20% acetonitrile: 80% water, injection volume: 1 μl. A Sciex QTrap 4500 with electrospray ionization source is used for the multiple reaction monitoring detection (Sciex). Mass spectrometer parameters for glucose assay were defined as follows: 179.6/89 Da pairs were used as Q1/Q3 for m +0, where ‘m’ denotes the ^12^C glucose mass to charge ratio, ‘+X’ denotes the number of ^13^C on glucose molecule. In similar fashion, 182/92 and 185/92 pair was used for m +3 and m +6, respectively. Electrospray ionization source parameters were set as follows: −4500V for negative mode, source temperature of 500 °C, curtain gas of 20 psi, ion source gas 1 and gas 2 of 50 to 60 psi, respectively, collusion energy of −14V, collusion exit potential of −11V.

### Immunoblotting

Flash-frozen liver tissue (100 mg) was homogenized in radioimmunoprecipitation assay buffer (50 mM Tris–HCl at pH 7.4, 150 mM NaCl, 1 mM EDTA, 1% Triton X-100, and 0.25% deoxycholate) with Roche PhosSTOP phosphatase inhibitor and protease inhibitor cocktail (Millipore Sigma). Homogenates were rotated for 30 min at 4 °C and then centrifuged at 18,000*g* for 15 min and supernatants isolated and transferred to new microcentrifuge tubes. Total protein concentrations were quantified by Pierce BCA assay (Thermo Fisher Scientific). Protein lysates (30ug input) were separated by Tris-glycine SDS-PAGE (4–15% polyacrylamide gels), followed by a transfer to Immobilon polyvinylidene difluoride membranes (Millipore Sigma). Membranes were blocked with 5% nonfat milk in Tris-buffered saline with Tween-20 (TBST) for 1 h and incubated overnight at 4 °C with primary antibodies at 1:1000 in 3% bovine serum albumin in TBST and all secondaries were diluted 1:5000 in 5% nonfat milk in TBST for 1 h. Antibodies used were as follows: pyruvate carboxylase (#16588-1-AP, Proteintech), HSC 70 (#sc-7298, Santa Cruz Biotechnology), pyruvate dehydrogenase (#2784 Cell Signaling), Citrate Synthase (#14309 Cell Signaling), Malate Dehydrogenase 2 (#11908 Cell Signaling), Mitochondrial Pyruvate Carrier 2 (17), Glutamine Oxaloacetate Transaminase 2 (HPA018139 Millipore Sigma), Carnitine Palmitoyltransferase 2 (PA5-12217 Thermo Fisher Scientific), Acyl-CoA Dehydrogenase Medium Chain (Genetex Irvine), Hydroxyacyl-CoA Dehydrogenase Trifunctional Multienzyme Complex Subunit Alpha (GTX101177 Genetex Irvine), Acyl-CoA Thioesterase 2 (15633-1-AP, Proteintech), Total Oxphos antibody cocktail (ab110411, Abcam), Acetylated-Lysine (#9441, Cell Signaling), mouse IgG-horseradish peroxidase (#7076, Cell Signaling), horseradish peroxidase–conjugated anti-rabbit (#NA934V Cytiva Life Sciences). Blots were imaged using the Amersham Prime enhanced chemiluminescent substrate (Cytiva Life Sciences) on an Alpha Innotech MultiImage III instrument. Biological replicates were used for all Western blots.

### Sample preparation for acetylome analysis of liver tissues from Pcx^L−/−^ mice

Liver tissues (50 mg) from Pcx^L−/−^ or WT mice (n = 5/biological replicates per genotype) were sonicated in lysis buffer (8 M urea, 50 mM triethylammonium bicarbonate [TEAB]) for 3 min. After centrifuging at 16,000*g* for 10 min, the supernatants were transferred to new tubes. The concentration of protein of the tissue lysates was measured by the Pierce BCA assay (Thermo Fisher Scientific). The protein lysates were reduced with 10 mM of tris (2-carboxyethyl) phosphine hydrochloride and 40 mM of chloroacetamide for 1 h at room temperature (RT). Two micrograms of protein lysate from each sample was digested with LysC (Wako Pure Chemical Industries, Ltd) at a ratio of 1:100 at 37 °C for 3 h. After dilution of urea from 8 M to 2 M urea by adding 50 mM TEAB, the protein lysate was digested with sequencing-grade trypsin (Promega) at a ratio of 1:50 at 37 °C overnight. After the solutions were acidified with 1% TFA, the digested peptides were desalted with Sep-Pak C_18_ light cartridges (Waters Corporation). Desalted peptides were lyophilized and reconstituted in 100 mM TEAB (pH 8.0) for TMT labeling. The order of the samples was randomized by an Excel function and then TMT labeling was carried out on them with 10-plex TMT reagents according to the manufacturer's instructions (Thermo Fisher Scientific). The peptides were reacted with TMT reagent for 1 h at RT and the reaction was quenched by 100 mM Tris–HCl (pH 8.0) for 20 min. TMT-labeled samples were pooled and lyophilized. The dried samples were reconstituted in 10 mM TEAB and high pH reversed-phase LC fractionation of the reconstituted sample was carried out using an Agilent Zorbax 300Extend-C18 HPLC column (4.6 mm × 25 cm, 5 μm; Agilent Technologies) on Agilent 1260 Infinity Capillary LC System (Agilent Technologies). The sample was separated into 96 fractions and they were concatenated into eight fractions followed by lyophilization.

Immunoaffinity purification (IAP) of acetyl peptides was carried out by using an Acetyl-Lysine Motif antibody kit (PTM Scan, Ac-K, Cell Signaling Technology) according to the manufacturer's instructions. Dried eight fractions were reconstituted with IAP buffer (PTM Scan, Cell Signaling Technology) and centrifugated at 12,000*g* for 5 min. Acetyl-Lysine Motif antibody beads were washed with IAP buffer three times before incubation. The supernatants were incubated with the washed antibody beads at 4 °C for overnight. After incubation, the beads were washed three times with IAP buffer and then twice with HPLC grade water. Lysine acetylated peptides were eluted with 55 μl and 50 μl of 0.15% TFA sequentially at RT for 10 min. Eluted peptides were desalted by homemade C_18_ Stage Tips.

### MS and data analyses

For the detection of posttranslationally modified proteins, the reconstituted samples (n = 5/biological replicates per genotype) in 0.1% formic acid were analyzed by Ultimate 3000 RSLCnano nano-flow LC coupled to Orbitrap Fusion Lumos Tribrid Mass Spectrometer (Thermo Fisher Scientific). The samples were loaded on an Acclaim PepMap100 Nano-Trap Column (100 μm × 2 cm; Thermo Fisher Scientific) with 5 μm C_18_ particles at a flow rate of 8 μl/min. Peptides were separated with a gradient of 8% to 28% solvent B (0.1% formic acid in 95% acetonitrile) at a 300 nl/min flow rate for 95 minutes on an EASY-Spray HPLC column (75 μm × 50 cm; Thermo Fisher Scientific) packed with 2 μm C_18_ particles. EASY-Spray ion source was operated at a voltage of 2.4 kV and the temperature of the ion transfer tube was set to 200  °C. The Orbitrap Fusion Lumos Tribrid Mass Spectrometer was operated in data-dependent mode with a full MS scan at the mass range 300 to 1800 at the top speed setting, 3 s per cycle. The precursor ions were measured in MS1 at a resolution of 120,000. Automatic gain control and maximum ion injection time for MS1 were set to 1,000,000 ions and 50 ms, respectively. The exclusion time of previously fragmented peptides was set to 30 s. Peptides were fragmented using higher energy collision dissociation and the normalized collision energy value was set at 35%. The first mass of MS/MS scans was set to 110 m/z. The fragmented peptide ions were measured in MS/MS at a resolution of 50,000. Automatic gain control and maximum ion injection time for MS/MS were set to 50,000 ions and 100 ms, respectively. The precursor isolation window was set to 1.6 m/z with a 0.4 m/z offset and single charged ions were rejected. Internal calibration was carried out using the lock mass option (m/z 445.12002).

Data analysis for identification and quantification of peptides was performed using Proteome Discoverer (v 2.4.1.15; Thermo Fisher Scientific). For MS/MS preprocessing, the top 10 peaks were selected within the 100 Da mass window. The MS/MS data were analyzed by SEQUEST algorithms with a mouse UniProt database released in December 2019 and common contaminant proteins database. The proteolytic enzyme was set to trypsin (full) with five maximum missed cleavage sites. The minimum and maximum peptide lengths were set to 6 and 35, respectively. Precursor mass tolerance was set to 10 ppm and fragment mass tolerance was set to 0.02 Da. Oxidation (+15.99492 Da) at methionine, acetylation (+42.01057 Da) at lysine and N-terminal, TMT 6-plex (+229.16293 Da) at lysine, Met-loss (−131.04048 Da) at methionine, and Met-loss+Acetyl (−89.02992 Da) at methionine were considered as a variable modification. TMT 6-plex (+229.16293 Da) at N-terminal and carbamidomethyl (+57.02146 Da) at cysteine were considered as a fixed modification. The minimum peptide length for the peptide filter was set to 6 aa and the minimum number of peptides per protein was set to 1. False-discovery rates (FDRs) for filtering peptides and peptides were set to 1% using a percolator node and the protein FDR validator node, respectively.

Quantification method was set to 10-plex TMT (channels: 126, 127N, 127C, 128N, 128C, 129N, 129C, 130N, 130C, 131). Peak integration tolerance was set to 20 ppm and the integration method was set to the most confident centroid. MS order was set to MS2. Both unique and razor peptides were used for peptide quantification. Protein groups were considered for peptide uniqueness. Shared quantification results were used. Missing values of intensity were replaced with the minimum value. A signal-to-noise ratio was used for computing reporter ion abundance. Quantification value corrections for isobaric tags were not applied. The coisolation threshold was set to 50% and the average reporter signal-to-noise threshold was set to 0. Data normalization was disabled. The strict parsimony principle was applied for protein grouping. All of the TMT reporter ion intensities of peptide-spectrum matches for the corresponding proteins were summed. The peptide table generated in Proteome Discoverer was exported, followed by importing it into Perseus 1.6.0.7 software (https://maxquant.net/perseus/) for normalization and statistical analysis. Acetyl-peptides were selected excluding nonacetyl peptides. The reporter ion intensities were normalized by dividing each row by the median value of the corresponding row, followed by dividing values in each column by the median value of nonacetyl peptides of the corresponding column. After normalization, the values in the table were log2-transformed. The log2 fold changes were calculated by subtracting the averages of the log2-transformed values between two groups. Student’s two-sample *t* test was performed for calculation of the *p* values. Significance analysis of microarrays and permutation-based FDR estimation calculation were performed for q values (32).

The COMPARTMENT dataset was used to assign localization for peptides for all cell compartments except mitochondria (33). Only COMPARTMENTS annotations with a minimum confidence score of 5 were used. Mitochondrial peptides were assigned using the MitoCarta 3.0 (34).

### Metabolites

Pcx^L−/−^ and Pcx^f/f^ mice were fasted at indicated time points and blood glucose and lactate levels were measured using a Nova Max Plus glucometer and Lactate Plus meter (Nova Biomedical). Blood was obtained by tail vain bleeds and collected using capillary tubes (Microvette CB 300, Sarstedt) and serum was isolated from blood following manufacturer’s instructions and stored at −80 °C. Serum TGs were analyzed using an Infinity Kit (Thermo Fisher Scientific). Serum cholesterol was assessed using a total Cholesterol E Kit (FUJIFILM Wako). Serum NEFAs were measured using a NEFA HR(2) kit (FUJIFILM Wako). Serum BHB was measured using a LiquiColor assay (Stanbio Laboratory). Biological replicates were used for all assays (n = 6–14/group).

### Liver metabolomics

Untargeted metabolomics was performed on fasted livers from Pcx^L−/−^ and Pcx^f/f^ mice (n = 6/biological replicates per genotype) by Metabolon Inc. For analysis, raw area counts for each biochemical species were rescaled to set the median equal to 1. Heatmap and principal component analysis were generated by MetaboAnalyst (35). Differentially regulated metabolites for heatmap were determined using a one-way ANOVA with Fisher’s LSD.

### Lipid extraction from liver tissue

Livers harvested from fed and 24 h fasted Pcx^L−/−^ and Pcx^f/f^ mice (n = 6/biological replicates per genotype) were collected, flash frozen in liquid nitrogen, and stored at −80 °C. About 100 mg of frozen liver tissue was homogenized in 500 μl of ice-cold molecular biology grade water (Quality Biological) as previously described (36). Next, 200 μl of the liver homogenate was collected for lipid extraction with 1 ml of chloroform:methanol (2:1) and centrifuged at 300*g* for 5 min at 4 °C. The lower chloroform phase was collected into a new microcentrifuge tube and dried in a vacuum. The dried lipid samples were resuspended in 50 μl of tert-butanol:MeOH:Triton-X100 (3:1:1) before determining TG content using an Infinity Kit (Thermo Fisher Scientific). Protein content of the liver homogenate was measured by using a Pierce BCA assay (Thermo Fisher Scientific) and lipid levels were normalized to total protein.

### RNA-seq library preparation and analysis

Total RNA was isolated from 100 mg flash-frozen liver tissue from Pcx^L−/−^ and Pcx^f/f^ mice (n = 4/biological replicates per genotype) using TRIzol reagent (Invitrogen for Thermo Fisher Scientific), followed by additional purification using RNeasy Mini Kit (QIAGEN), per manufacturer recommendations. RNA quality was assessed by Nanodrop. RNA was then submitted to Novogene Corporation Inc (China & Davis) for library construction and sequencing. Four biological replicates were used for each genotype. Reads were aligned to mouse reference genome (mm10).

Differential expression was performed on raw read counts in R with DESeq2 (v3.12) using the Wald test with betaPrior = FALSE and lfcShrink type=”apeglm” (37, 38). Differential expression cutoff was fold change ≥ |2|, padj < 0.05.

### Quantitative real-time PCR analysis

Total RNA was isolated from 100 mg of flash-frozen liver from Pcx^L−/−^ and Pcx^f/f^ mice (n=5–7/biological replicates per genotype) as described previously for RNA-seq preparation by TRIzol reagent. RNA samples were subjected to DNASE I (New England Biolabs) digestion to remove genomic DNA contamination using the manufacturer's protocol. A 3 M (pH 5.2) sodium acetate (Quality Biological) precipitation was performed after DNASE I digestion to purify RNA samples. In brief, sodium acetate was added at 1/10 the volume of the sample followed by the addition of 2.5 volumes of ice cold 100% ethanol and mixed thoroughly. The samples were incubated at −80 °C overnight. The samples were centrifuged at 12,000*g* for 20 min at 4 °C and the supernatant was decanted. The RNA pellets were washed twice using 1 ml of ice cold 75% ethanol and centrifuged at 12,000*g* at 4 °C. A quick spin was performed to remove any traces of residual ethanol in the pellet using a very fine pipette tip. The RNA pellets were air dried and reconstituted in sterile filtered molecular biology grade water (Quality Biological). Following RNA quality assessment by Nanodrop, complementary DNA (cDNA) was synthesized from 2 μg RNA using the High Capacity cDNA Reverse Transcription Kit (Applied Biosystems for Thermo Fisher Scientific). Quantitative real-time PCR analyses were carried out on 10 ng of cDNA using SsoAdvanced Universal SYBR Green Supermix (Bio-Rad) per manufacturer recommendations. RNA was first normalized to 18S ribosomal RNA to generate a ΔC_t_ value and then ΔΔC_t_ was obtained by normalizing data to mean ΔC_t_ of the control group (39). Primers used are listed in Table S1.

### Statistical analysis

All statistical comparisons were carried out in GraphPad Prism 9 (GraphPad Software Inc) unless otherwise noted. Significance was determined using Student’s *t* test, one-way ANOVA with Tukey’s post hoc correction. For experiments with two independent variables, a two-way ANOVA was conducted and the Bonferroni method was used for multiple comparisons. Shapiro–Wilk test for normality was used in R to determine whether to use a parametric or nonparametric test for significance for genomic data. Values were considered significant at *p* < 0.05.

## Data availability

RNA-seq data were deposited in GSE198317.

All proteomic data were deposited in the Proteomics Identifications Database #PXD032028.

## Supporting information

This article contains supporting information.

## Conflict of interest

The authors declare that they have no conflicts of interest with the contents of this article.
