# Supplementary material for: Requirement of hepatic pyruvate carboxylase during fasting, high fat, and ketogenic diet
Source: J Biol Chem. 2022 Oct 28;298(12):102648. doi: 10.1016/j.jbc.2022.102648 (PMC9694104; doi:10.1016/j.jbc.2022.102648)
Supplement: Supporting information [file mmc5.docx]

**SUPPORTING TABLES**

**Supporting Table 1:** Primer Table

**Supporting Table 2:** Unbiased liver metabolomics of 24hr fasted Pcx^ff^ and Pcx^L-/-^ mice.

**Supporting Table 3:** RNA-seq analysis of 24hr fasted Pcx^ff^ and Pcx^L-/-^ mice.

**Supporting Table 4:** Subcellular Acetyl-proteomic analysis of 24hr fasted Pcx^ff^ and Pcx^L-/-^ mice.

**SUPPORTING FIGURES**

**Supporting Figure 1. The role of high fat feeding on female liver specific pyruvate carboxylase knockout mice.**

1. Body weight gain of female Pcx^f/f^ and Pcx^L-/-^ mice fed a high fat diet for 12 weeks.
2. Weight of gWAT, iWAT, kidney and liver of female Pcx^f/f^ and Pcx^L-/-^ mice fed a high fat diet for 12 weeks.
3. Blood glucose, triglyceride, cholesterol, NEFA and beta-hydroxybutyrate in the fed state of female Pcx^f/f^ and Pcx^L-/-^ mice fed a high fat diet for 12 weeks.

Data are expressed as mean ± SEM. *p<0.05; **p<0.01; ***p<0.001.

**Supporting Figure 2. Liver specific pyruvate carboxylase knockout female mice fed a high fat diet.**

1. Fasted blood glucose, lactate, and cholesterol, of female Pcx^f/f^ and Pcx^L-/-^ mice fed a high fat diet for 12 weeks.
2. Fasted blood beta-hydroxybutyrate, NEFA, and triglyceride, of female Pcx^f/f^ and Pcx^L-/-^ mice fed a high fat diet for 12 weeks.
3. Intraperitoneal glucose tolerance tests of female Pcx^f/f^ and Pcx^L-/-^ mice fed a high fat diet for 12 weeks.
4. Area under the curve of glucose tolerance test of female Pcx^f/f^ and Pcx^L-/-^ mice fed a high fat diet for 12 weeks.
5. Insulin tolerance tests of female Pcx^f/f^ and Pcx^L-/-^ mice fed a high fat diet for 12 weeks.
6. Area under the curve of insulin tolerance test of female Pcx^f/f^ and Pcx^L-/-^ mice fed a high fat diet for 12 weeks.

Data are expressed as mean ± SEM. *p<0.05; **p<0.01; ***p<0.001.

**Supporting Figure 3. A ketogenic diet causes metabolic decompensation in female liver specific pyruvate carboxylase knockout mice.**

1. Body weight of 9 week old female Pcx^f/f^ and Pcx^L-/-^ mice before and after feeding a ketogenic diet for 1 week.
2. Blood glucose of 9 week old female Pcx^f/f^ and Pcx^L-/-^ mice before and after feeding a ketogenic diet for 1 week.
3. Blood lactate of 9 week old female Pcx^f/f^ and Pcx^L-/-^ mice before and after feeding a ketogenic diet for 1 week.
4. Blood beta-hydroxybutyrate, NEFA, triglyceride and cholesterol of of 9 week old female Pcx^f/f^ and Pcx^L-/-^ mice after feeding a ketogenic diet for 1 week.
5. Kidney and liver weights of 9 week old female Pcx^f/f^ and Pcx^L-/-^ mice after feeding a ketogenic diet for 1 week.

Data are expressed as mean ± SEM. *p<0.05; **p<0.01; ***p<0.001.
