# Supplementary material for: Requirement of hepatic pyruvate carboxylase during fasting, high fat, and ketogenic diet
Source: J Biol Chem. 2022 Oct 28;298(12):102648. doi: 10.1016/j.jbc.2022.102648 (PMC9694104; doi:10.1016/j.jbc.2022.102648)

Supporting Figure 1.

The role of high fat feeding on female liver specific pyruvate carboxylase knockout mice.

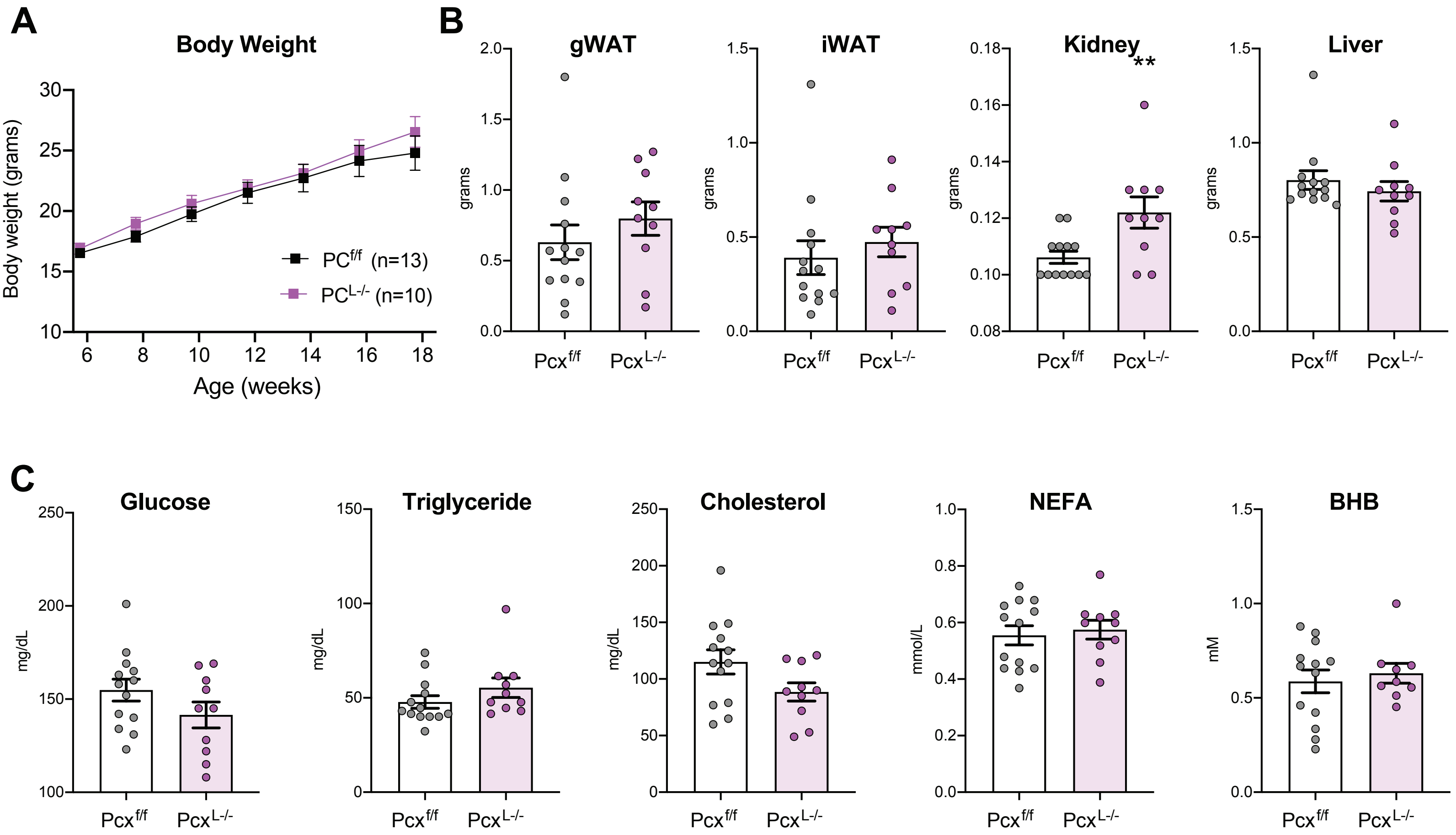

Supplement: Figure S1 [file mmc6.pdf]
