# Supplementary material for: Requirement of hepatic pyruvate carboxylase during fasting, high fat, and ketogenic diet
Source: J Biol Chem. 2022 Oct 28;298(12):102648. doi: 10.1016/j.jbc.2022.102648 (PMC9694104; doi:10.1016/j.jbc.2022.102648)

Supporting Figure 2.

Liver specific pyruvate carboxylase knockout female mice fed a high fat diet.

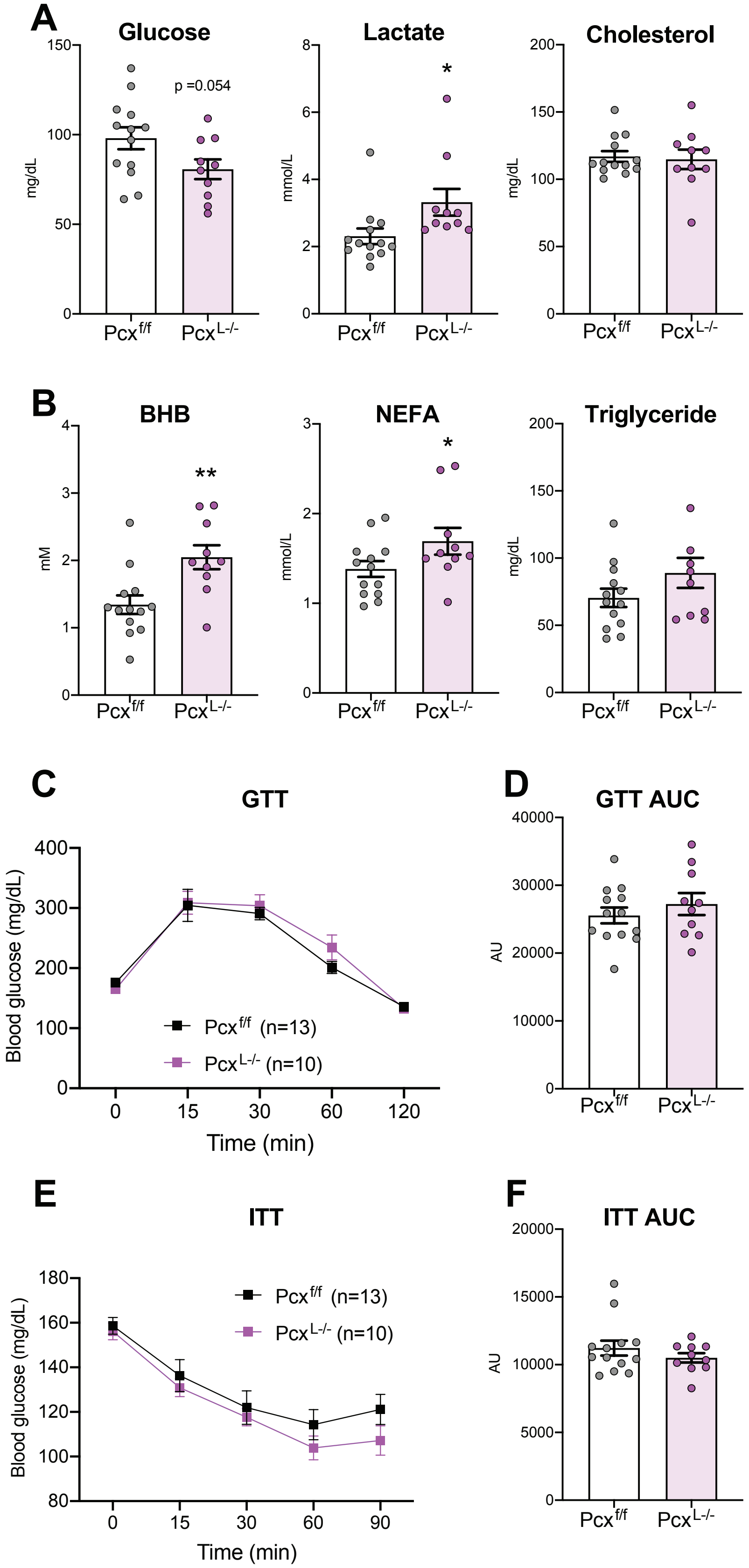

Supplement: Figure S2 [file mmc7.pdf]
